# In Vivo Anti-Inflammatory Potential of Viscozyme^®^-Treated Jujube Fruit

**DOI:** 10.3390/foods9081033

**Published:** 2020-08-01

**Authors:** Yoonsu Kim, Jisun Oh, Chan Ho Jang, Ji Sun Lim, Jeong Soon Lee, Jong-Sang Kim

**Affiliations:** 1 School of Food Science and Biotechnology (BK21PLUS), Kyungpook National University, Daegu 41566, Korea; yunsu531@gmail.com (Y.K.); cksghwkd7@gmail.com (C.H.J.); 2 Institute of Agriculture Science and Technology, Kyungpook National University, Daegu 41566, Korea; j.oh@knu.ac.kr (J.O.); lzsunny@daum.net (J.S.L.); 3 Forest Resources Development Institute of Gyeongsangbuk-do, Andong 36605, Korea; ljs7942@korea.kr

**Keywords:** Jujube, hydrolysis, anti-inflammation, lung, NF-κB, Nrf2, HO-1

## Abstract

The fruit of *Ziziphus jujuba*, commonly called jujube, has long been consumed for its health benefits. The aim of this study was to examine the protective effect of dietary supplementation of enzymatically hydrolyzed jujube against lung inflammation in mice. The macerated flesh of jujube was extracted with aqueous ethanol before and after Viscozyme treatment. The extract of enzyme-treated jujube, called herein hydrolyzed jujube extract (HJE), contained higher levels of quercetin, total phenolics, and flavonoids, and exhibited more effective radical-scavenging abilities in comparison to non-hydrolyzed jujube extract (NHJE). HJE treatment decreased production of inflammation-associated molecules, including nitric oxide and pro-inflammatory cytokines from activated Raw 264.7 or differentiated THP-1 cells. HJE treatment also reduced expression of nuclear factor-κB and its downstream proteins in A549 human lung epithelial cells. Moreover, oral supplementation of 1.5 g of HJE per kg of body weight (BW) attenuated histological lung damage, decreased plasma cytokines, and inhibited expression of inflammatory proteins and oxidative stress mediators in the lungs of mice exposed to benzo(*a*)pyrene at 50 mg/kg BW. Expression levels of antioxidant and cytoprotective factors, such as nuclear factor erythroid-derived 2-related factor 2 and heme oxygenase-1, were increased in lung and liver tissues from mice treated with HJE, compared to mice fed NHJE. These findings indicate that dietary HJE can reduce benzo(*a*)pyrene-induced lung inflammation by inhibiting cytokine release from macrophages and promoting antioxidant defenses in vivo.

## 1. Introduction

Lung inflammation can be triggered by pathogens, toxins, and pollutants [1,2,3]. In response to exogenous inflammatory factors, the innate immune defense system is acutely activated to rehabilitate affected tissue and minimize systemic inflammation [4]. If the inflammatory response is not sufficient to restore tissue homeostasis, lung damage progresses and causes pulmonary dysfunction and respiratory failure [5]. 

Inflammation in the lungs is regulated by a variety of molecular mediators, including pro- and anti-inflammatory chemokines and cytokines, which are produced by damaged pulmonary epithelial cells, as well as various types of activated leukocytes [6,7]. Mediators related to lung inflammation include tumor necrosis factor α (TNFα), interleukin-6 (IL-6), and interleukin-1β (IL-1β), which regulate maturation of dendritic cells and induce recruitment of neutrophils at inflammatory sites [7,8]. Cyclooxygenase (COX) is an enzyme that metabolizes arachidonic acid into endoperoxides and is closely associated with inflammation [9]. Of the two isoforms, COX-2 is expressed in most cells in an inducible manner at the local inflammation site, whereas COX-1 is constitutively expressed. COX-2 expression is controlled by nuclear factor κB (NF-κB) which is a ubiquitous transcription factor that regulates expression of multiple proteins involved in inflammatory and immune responses [10].

Nuclear factor erythroid-derived 2-related factor 2 (Nrf2) is a key regulator of a set of phase-2 defense and antioxidant enzymes, including heme oxygenase-1 (HO-1) [11]. Nrf2 and its downstream enzymes play a protective role against pro-inflammatory and detrimental oxidizing effects [12]. Several studies have demonstrated that antioxidative phytochemicals in fruits and vegetables can upregulate the Nrf2 signaling pathway, downregulate the NF-κB pathway, and consequently modulate inflammation-associated gene expression in macrophages and lung epithelial cells [13,14].

The fruit of *Ziziphus jujuba* var. inermis Rehder (commonly called jujube) is used widely as a dietary ingredient and traditional remedy for digestive and respiratory dysfunctions [15,16,17,18]. Jujube contains not only antioxidative phytochemicals, including phenolics and flavonoids, but also polysaccharides with immunomodulatory potential [19,20,21]. These compounds were found to exert protective effects against gastrointestinal, hepatic, and pulmonary damage [19,20,21]. A couple of studies have demonstrated that crude mixtures containing jujube extract can alleviate symptoms of nasal or gastrointestinal inflammation in mice [22,23]. Some polysaccharides obtained from jujube by ultrasonic-assisted extraction reportedly suppressed pro-inflammatory cytokine production in activated Raw 264.7 cells [24,25]. In addition, a research group has shown that jujuboside B, a saponin found in jujube ethanolic extract, lowered the number of immune cells in bronchoalveolar lavage fluid, and decreased levels of allergic phenotype-regulating cytokines in lung homogenate from an ovalbumin-induced allergic asthma mouse model [26]. These findings suggest that jujube ethanolic extract contains a variety of bioactive substances and may relieve lung disease caused by exogenous inflammatory stimuli.

In an attempt to liberate the bioactive components existing in inactive glycoside forms, jujube fruit was preheated and enzymatically treated using Viscozyme, which possesses primarily endo-1,3(4)-β-glucanase and collaterally cellulase and hemicellulase activities, to break any constraining linkage and thereby enhance the bioavailability of the compounds. The fruit was then subjected to extraction with aqueous ethanol. The hydrolyzed jujube fruit extract was evaluated for its biologically beneficial effects in an acute lung inflammation mouse model using benzo(*a*)pyrene (B(a)P).

## 2. Materials and Methods

### 2.1. Preparation of Hydrolyzed Jujube Extracts

The commercially available fresh jujube fruits that were harvested from Gyeongsan region, S. Korea in October 2019 [16,27] were obtained from a local market. The seeds were removed and the flesh was cut into 100 g samples. The samples were homogenized with 10 volumes of distilled water, pretreated at 95 °C for 10 min, followed by enzymatic hydrolysis using five units of Viscozyme (Novozyme, Bagsvaerd, Denmark) in 1 M acetate buffer at a pH of 5.0 and a temperature of 55 °C, shaken at 250 rpm for 1 h. Non-hydrolyzed jujube flesh homogenate was subjected to the same procedure as HJE but without enzyme treatment. Both samples were then mixed with the same volume of absolute ethanol to make the final concentration of 50% (*v/v*) ethanol in solution, and extracted in a shaking incubator at 55 °C and 250 rpm for 1 h. Both extracts prepared in the absence and presence of Viscozyme were referred to as non-hydrolyzed jujube extract (NHJE) and hydrolyzed jujube extract (HJE), respectively. They were filtered using 8-μm filter papers (Whatman, Little Chalfont, UK), vacuum-evaporated (EYELA N-1000, Tokyo, Japan), and freeze-dried. The NHJE and HJE powders were dissolved in dimethyl sulfoxide (DMSO) at the concentration of 500 mg/mL for further experiments.

### 2.2. Quantification of Total Phenolics and Flavonoids

Total phenolic and flavonoid contents in the jujube extracts were determined as reported previously [28,29,30]. For quantification of the total phenolic content, briefly, each sample was mixed with Folin reagent and 10% sodium carbonate. After incubation at room temperature for 1 h, the absorbance was measured at 725 nm using a microplate reader. For measurement of the total flavonoid content, samples were mixed with aluminum nitrate, potassium acetate, and ethanol. After incubation at room temperature for 40 min, the absorbance was acquired at 415 nm. Total phenolic compound content and total flavonoid content were estimated from a standard curve prepared using gallic acid and quercetin (both from Sigma-Aldrich, St. Louis, MO, USA), respectively.

### 2.3. High-Performance Liquid Chromatography Analysis

Quantitative determination of rutin and quercetin in NHJE and HJE was performed using high-performance liquid chromatography (Shimadzu Corporation, Tokyo, Japan) equipped with a photodiode array (PDA) detector and an M1 column (SunFire, C18, 4.6 × 250 mm, 5-μm particle size) with the mobile phase consisting of a mixture of 40% (*v/v*) methanol and 15% (*v/v*) acetonitrile in water containing 1% (*v/v*) acetic acid. The flow rate was 1 mL/min. The column temperature was maintained at 40 °C for 30 min during the analysis and the injection volume was 10 μL. Detection was made with a PDA detector at 280 nm. The quantities of those compounds were determined by extrapolating the corresponding peak areas from the calibration curves of standards, rutin, and quercetin (both from Sigma-Aldrich).

### 2.4. Cell Culture and Treatment

Raw 264.7 murine macrophages, THP-1 human monocytes, and A549 human lung epithelial cells were obtained from the Korean Cell Line Bank (Seoul, Korea). All cell lines were grown in Dulbecco’s modified Eagle medium (DMEM, Invitrogen) containing 10% heat-inactivated fetal bovine serum (FBS) and 1% penicillin-streptomycin (Welgene, Gyeongsan, Gyeongbuk) and placed in a humidified incubator that provided 5% CO_2_ at 37 °C.

For cell viability assays, cells were dispensed into a 96-well plate at a density of 5 × 10^3^ cells/well, treated with jujube extract at the concentrations of 0, 62.5, 125, 250, and 500 μg/mL for 24 h, and assayed using the Cell Counting Kit (CCK-8; Dojindo Laboratories, Kumamoto, Japan) as previously described [31].

For measurement of nitric oxide (NO) levels in culture medium, Raw 264.7 cells were seeded at a density of 2 × 10^3^ cells per well of a 96-well plate and activated by treatment with lipopolysaccharide (LPS) at 1 μg/mL for 24 h. The culture medium was collected and reacted with the Griess reagent (Promega, Madison, WI, USA).

THP-1 cells were passaged at a density of 4 × 10^6^ cells/mL in a 100-mm plate and maintained in DMEM containing 10% FBS. The cells were activated and differentiated by applying 200 nM of 12-*O*-tetradecanoylphorbol-13-acetate (TPA). For indirect co-culture of THP-1 and A549 cells, the cultured medium (herein called ‘conditioned medium’) from THP-1 cells was collected and transferred to A549 cells.

### 2.5. Antioxidant Response Element–Luciferase Reporter Assay

To determine the transcriptional activity of the antioxidant response element (ARE), a luciferase reporter gene assay was conducted on a HepG2 cell line carrying the ARE-luciferase construct using a luciferase assay system (Promega, Madison, WI, USA) [32]. Sulforaphane (Sigma-Aldrich, St. Louis, MO, USA) was used as a positive control.

### 2.6. Animal Treatment

The animal study was approved by, and conducted according to the guidelines of, the Institutional Animal Care and Use Committee of the Kyungpook National University (approval number: KNU 2019-0157). A total of 48 adult C57BL/6J mice (six weeks old males, 20−25 g BW) were purchased from Hyochang Science (Daegu, Korea). After one week of adaptation in a controlled laboratory environment (temperature, 22 ± 2 °C; humidity, 50 ± 5%; 12-h light-dark cycle) with free access to water and AIN-76A chow (Hyochang Science, Daegu, Korea), the mice were randomly assigned to six groups (eight mice per group). The experimental groups were as follows: (1) control, a group treated only with vehicle, (2) a group treated with B(a)P alone, (3) a group treated with NHJE at 0.75 g/kg BW and B(a)P, (4) a group treated with NHJE at 1.5 g/kg BW and B(a)P, (5) a group treated with HJE at 0.75 g/kg BW and B(a)P, and (6) a group treated with HJE at 1.5 g/kg BW and B(a)P. The NHJE and HJE samples were dissolved in a vehicle composed of 5% (*v/v*) Tween-80, 5% ethanol, and 90% sterilized drinking water. The freshly dissolved samples were administered by oral gavage in a volume of 0.2 mL/mouse on a daily basis for two weeks. Lung inflammation was induced by intraperitoneal injection with 50 mg/kg BW of B(a)P dissolved in corn oil (Sigma-Aldrich, St. Louis, MO, USA) 24 h prior to sacrifice. The blood, lung, and liver tissues were dissected and used for further biochemical analyses.

### 2.7. Histopathological Analysis

The entire lungs were dissected from the sacrificed. All lobes were fixed in a 10% (*v/v*) formalin solution and embedded in paraffin [33]. Tissue blocks were then coronally sectioned into 5-μm-thick slices using a microtome (RM-2125 Rt; Leica, Nussloch, Germany), and stained with hematoxylin and eosin (H&E, Sigma-Aldrich, St. Louis, MO, USA). Histological damage in the tissue sections was observed under a microscope (Eclipse 80i; Nikon, Tokyo, Japan).

### 2.8. Enzyme-Linked Immunosorbent Assay

An enzyme-linked immunosorbent assay (ELISA) was performed using commercially available kits for the quantitation of inflammatory cytokines (TNFα, IL-1β, and IL-6) in plasma (mouse ELISA sets for each cytokine, BD Biosciences, San Jose, CA, USA). Lung and liver tissues were collected from the mice and homogenized in phosphate buffered saline including protease inhibitor cocktail (Roche, Mannheim, Germany). After centrifugation at 15,000× *g* for 10 min, the supernatants were used for the following analyses. Lung tissue homogenate was subjected to measurement of IL-10 (ELISA MAX standard set, Biolegend, San Diego, CA, USA) and prostaglandin E_2_ (PGE_2_) (Cayman Chemical, Ann Arbor, MI, USA) as per the manufacturer’s instructions. Liver tissue homogenate was used to determine the levels of 8-hydroxy-2′-deoxyguanosine (8-OHdG) (Cat # ADI-EKS-350; Enzo Life Sciences, Farmingdale, NY, USA) and malondialdehyde (MDA) (Cat # ALX-850-287; Enzo Life Sciences, Inc., Farmingdale, NY, USA) as previously described [34]. The obtained values were normalized to the total amount of proteins.

### 2.9. Western Blot Analysis

Cytoplasmic and nuclear fractions of cultured cells and mouse tissues were prepared using the NE-PER Nuclear and Cytoplasmic Extraction Reagents (Thermo Fisher Scientific, Rockford, IL, USA). The proteins in each fraction were blotted and their relative expression levels were determined as previously described [34,35]. Briefly, the primary antibodies used in this study were immunoglobulins against COX-2 (Cell Signaling Technology, Danvers, MA, USA), *NF*-*κB* (Bioworld Technology, St. Luis. MN, USA), iNOS (Enzo Life Sciences, Farmingdale, NY, USA), HO-1 (Abcam, Cambridge, UK), Nrf2 (Abcam, Cambridge, UK), β-actin, and Lamin B1 (Santa Cruz Biotechnology, Dallas, TX, USA). They were used at a dilution ratio of 1:1000 in 1% bovine serum albumin (Bio Basic Inc., Markham, ON, Canada) in tris-buffered saline (TBS). After allowing the appropriate secondary antibody (horse radish peroxidase–conjugated) at 1:2000 in TBS to interact with the primary antibody, protein bands were visualized using SuperSignal West Pico Chemiluminescent Substrate (Pierce, Cheshire, United Kingdom) and LAS4000 Mini (GE Healhcare Life Sciences, Little Chalfont, UK). The digitalized blot images were then densitometrically analyzed using Image-Studio Lite version 5.2 (LI-COR Biotechnology, Lincoln, NE, USA).

### 2.10. Statistical Analysis

All statistical analyses were performed using SPSS version 23.0 (SPSS Inc., Chicago, IL, USA). Statistical differences among means were tested by paired *t*-tests or one-way analysis of variance followed by Duncan’s multiple range test. The *p* values less than 0.05 or 0.1 were considered significant. Different alphabetical letters indicate statistically significant difference between values.

## 3. Results

### 3.1. Enzyme Hydrolysis of Jujube Fruit Increased Total Phenolic Content in Ethanolic Extract

For the preparation of HJE, the concentration of ethanol in the extraction solvent was determined to be 50% (*v/v*) in water, based on the contents of total phenolics and flavonoids that were potent bioactive compounds (Supplementary Table S1). For hydrolysis of jujube fruit, Viscozyme was chosen among multiple food industrial enzymes as the highest total phenolic content was acquired in a 50% ethanol extract after enzymatic reaction (Supplementary Table S2). HJE exhibited higher antioxidant capacities assessed by a 2,2-diphenyl-1-picrylhydrazyl radical-scavenging assay and ferric reducing antioxidant power values (Supplementary Figure S1), and also contained approximately 1.9-fold higher total phenolic content and 10.9-fold higher quercetin content than its non-hydrolyzed counterpart, NHJE (Table 1).

### 3.2. HJE Decreased NO Production and Increased ARE Transcription Activity in Cultured Cells

**Figure 1:**
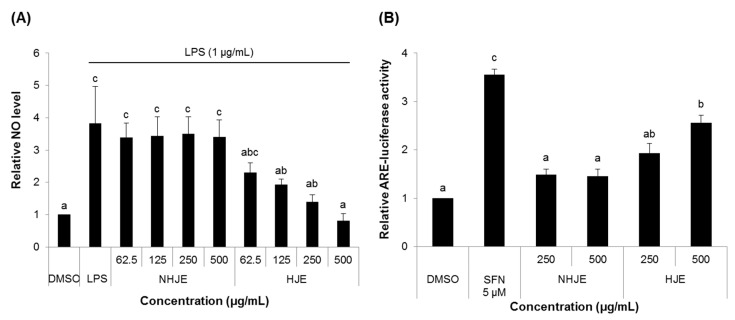
HJE decreased nitric oxide (NO) production and increased antioxidant response element (ARE) transcription activity in cultured cells. (**A**) Raw 264.7 cells were stimulated by LPS and treated with NHJE and HJE for 24 h. The culture media were collected and measured for the levels of NO produced from the cells. (**B**) HepG2-ARE cells carrying the luciferase reporter gene linked to the ARE sequence were treated with NHJE and HJE for 24 h. ARE transcription activity was assessed by the activity of luciferase. SFN, sulforaphane. *N* = 3; error bars, mean ± SEM. Different alphabetical letters on the bars (a–c) indicate statistically significant difference from each other (*p* < 0.05).

The anti-inflammatory and antioxidant effects of HJE and NHJE were compared in both Raw 264.7 cells and HepG2-ARE cells at concentrations of ≤500 µg/mL, which were non-toxic to both cell lines (Supplementary Figure S2). HJE decreased production of NO in a dose-dependent manner in LPS-stimulated Raw 264.7 cells (Figure 1A) and significantly increased ARE transcription activity in HepG2-ARE cells (Figure 1B), whereas treatment with NHJE had no significant effect on NO levels and ARE-transcriptional activity of these cells.

### 3.3. HJE Inhibited Secretions of Pro-Inflammatory Cytokines from TPA-Challenged THP-1 Cells and Expressions of Inflammation-Related Proteins in A549 Cells

To investigate the role of HJE in interactions between macrophages and lung epithelial cells, human monocyte THP-1 cells were differentiated by TPA treatment and incubated in the absence or presence of either HJE or NHJE, and the resulting cultured media were added to lung epithelial A549 cell culture, followed by measurements of the expression of pro-inflammatory proteins, such as iNOS, COX-2, and NF-κB.

**Figure 2:**
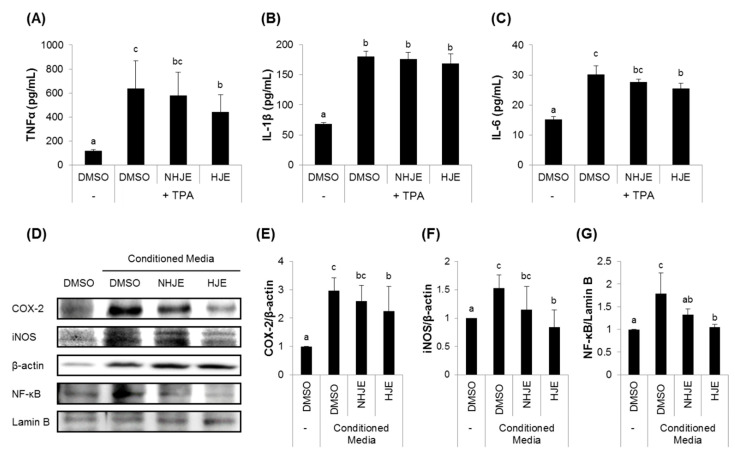
HJE decreased secretions of pro-inflammatory cytokines from differentiated THP-1 cells and expression of inflammation-related proteins in A549 cells. (**A**–**C**) THP-1 human monocytes were differentiated with tetradecanoylphorbol-13-acetate (TPA) (200 nM) and treated with either NHJE or HJE at a concentration of 500 µg/mL for 24 h. The pro-inflammatory cytokines, including TNFα (**A**), IL-1β (**B**), and IL-6 (**C**), in the culture media were quantified by enzyme-linked immunosorbent assay (ELISA). (**D**–**G**) A549 human lung epithelial cells were cultured in conditioned media collected from TPA-differentiated THP-1 cells treated with either NHJE or HJE. After 24 h, A549 cells were collected and subjected to western blot analysis (**D**). The relative expression levels of inflammation-related proteins, including COX-2 (**E**), iNOS (**F**), and NF-κB (**G**), were densitometrically determined. *N* = 3; error bars, mean ± SEM. Different alphabetical letters (a–c) presented on the bars indicate statistically significant difference from each other (*p* < 0.05).

Quantification of pro-inflammatory cytokines in the THP-1-cultured medium (the conditioned medium) revealed that HJE considerably reduced production of TNFα and IL-6 from THP-1 cells, while NHJE had no effect (Figure 2A−C). A549 cells maintained in the conditioned media from HJE-treated THP-1 cells expressed relatively lower levels of NF-κB, cytoplasmic iNOS and COX-2 proteins compared with the cells kept in NHJE-treated conditioned media (Figure 2D–G).

### 3.4. Oral Supplementation of HJE Alleviated B(a)P-Induced Lung Injury in Mice

**Figure 3:**
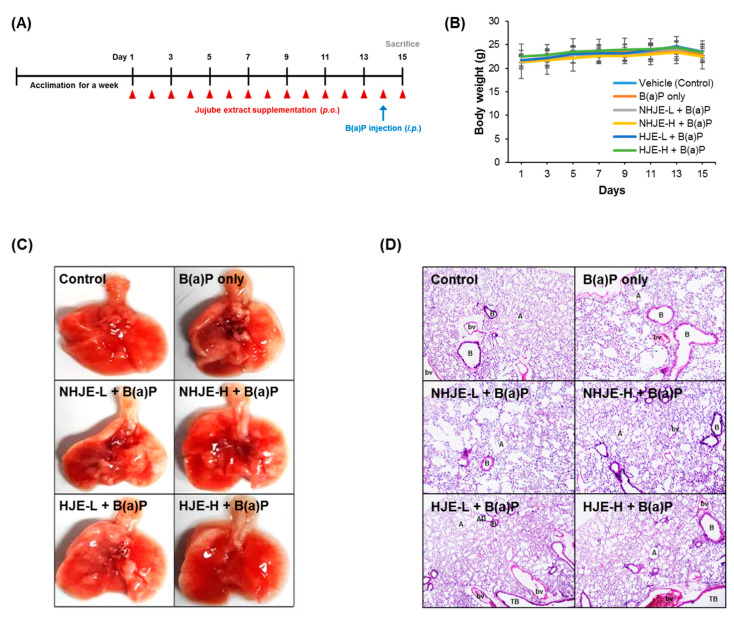
Oral administration of HJE alleviated B(a)P-induced lung injury in mice. C57BL/6J mice were fed either NHJE or HJE at two different doses, 0.75 g/kg BW or 1.5 g/kg BW, every day for 14 days. One day prior to sacrifice, B(a)P was given by intraperitoneal injection. (**A**) Experimental scheme. (**B**) Changes in average body weight during the experimental period. Values are means ± standard deviation (SD) (*n* = 8 mice per group). (**C**) Representative photographs of the dissected lungs. (**D**) Representative tissue sections stained with H&E (magnification, 40×). A, alveolar sac; AD, alveolar duct; B, respiratory bronchiole; TB, terminal bronchiole; bv, blood vessel. NHJE, non-hydrolyzed jujube extract; NHJE-L, NHJE at a low dose (0.75 g/kg BW); NHJE-H, NHJE at a high dose (1.5 g/kg BW); HJE, hydrolyzed jujube extract; HJE-L, HJE at a low dose (0.75 g/kg BW); HJE-H, HJE at a high dose (1.5 g/kg BW).

To further evaluate the anti-inflammatory effect of HJE in vivo, acute lung inflammation in mice was induced by a single intraperitoneal injection of B(a)P 24 h prior to sacrifice after 14 days of oral administration of either NHJE or HJE (Figure 3A). The changes in the average BW of mice were insignificant among the groups during the experimental period (Figure 3B). Histological observation of lungs indicated that supplementation with HJE or NHJE at a high dose (1.5 g/kg BW) attenuated B(a)P-induced lung injury (Figure 3C). In addition, H&E-stained lung tissue sections displayed that B(a)P treatment caused disruption of lung architecture [36,37,38], including poor arrangement of epithelial cells lining bronchiole and abnormal morphology of alveolar structure (inflated alveolar sacs and thickened or ruptured interalveolar septa). However, the severity of B(a)P-induced histological damage in the lungs was reduced in mice supplemented with HJE or NHJE-H (Figure 3D).

### 3.5. Oral Supplementation of HJE Lowered Plasma Pro-Inflammatory Cytokine Levels in B(a)P-Injected Mice

**Figure 4:**
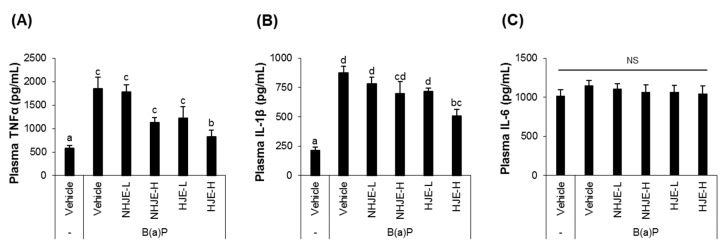
Oral administration of HJE lowered plasma levels of pro-inflammatory cytokines in B(a)P-injected mice. (**A**–**C**) Levels of pro-inflammatory cytokines, TNFα (**A**), IL-1β (**B**), and IL-6 (**C**), in the mouse blood samples were measured by ELISA. Values are means ± SD (*n* = 8). Different alphabetical letters presented on the bars (a–d) indicate statistically significant difference from each other (*p* < 0.05). NS, not significant.

After supplementation with the extract for 14 days and then intraperitoneal injection with B(a)P, blood samples were collected from mice and subjected to ELISA for quantification of TNFα, IL-1β, and IL-6 (Figure 4). The group fed HJE at a high dose (1.5 g/kg BW) showed significantly lower levels of TNFα and IL-1β in plasma, compared with the group injected with B(a)P without jujube extract supplementation.

### 3.6. Oral Supplementation of HJE Suppressed the Expression of Inflammation-Related Proteins and Increased the Expression of Antioxidant Proteins in Lung Tissue

**Figure 5:**
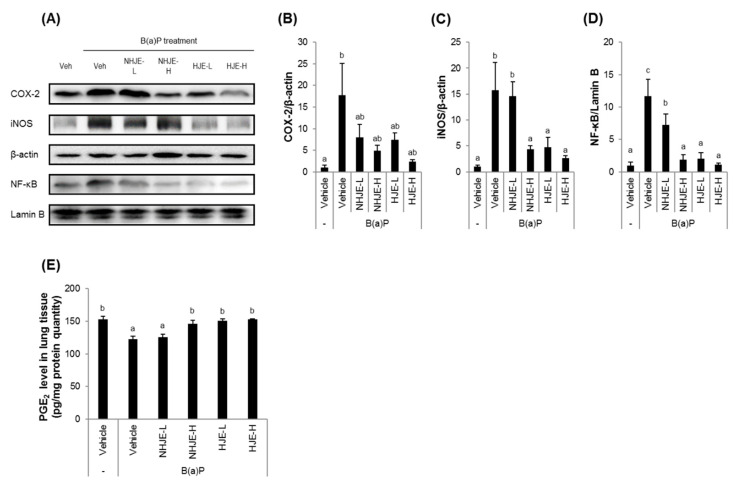
Oral administration of HJE suppressed expression of inflammation-related proteins in lung tissue. Expression levels of inflammation marker proteins in the lung tissues were examined. (**A**) Representative western blot images. (**B**–**D**) Quantitative data for cytosolic COX-2 (**B**), cytosolic iNOS (**C**), and nuclear NF-κB (**D**). (**E**) PGE_2_ protein levels in dissected lung tissues were quantified using ELISA. Values are mean ± SD (*n* = 5). Different alphabetical letters on the bars (a–c) indicate statistically significant difference from each other (*p* < 0.05).

Lung tissue homogenates were analyzed for the expression levels of inflammatory proteins, such as NF-κB, cytoplasmic iNOS, and COX-2 (Figure 5A−D). Treatment with HJE (0.75 and 1.5 g/kg BW) or NHJE (1.5 g/kg BW) significantly suppressed protein expression of NF-κB, cytoplasmic iNOS, and COX-2, which were increased by B(a)P injection. Moreover, the tissue level of PGE_2_, which limits inflammation and promotes lung tissue repair [39], was reduced in lungs of mice treated with B(a)P alone but restored by co-treatment with 0.75 g HJE/kg BW or 1.5 g NHJE/kg BW, indicating that HJE is more effective than NHJE (Figure 5E).

**Figure 6:**
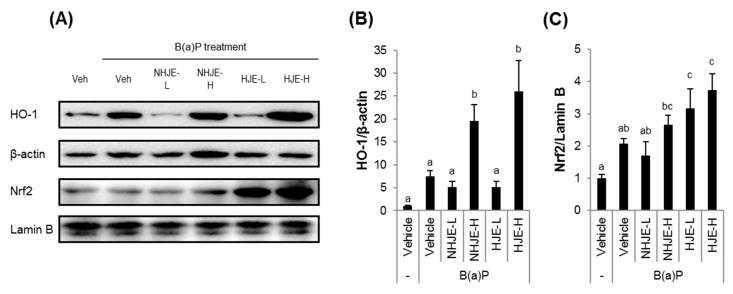
Oral administration of HJE increased Nrf2 and HO-1 expressions in lungs. Expression levels of a key antioxidant/defense transcription factor Nrf2 and its downstream protein HO-1 in the lung tissues were examined. (**A**) Representative western blot images. (**B**–**C**) Quantitative data for cytosolic HO-1 (**B**) and nuclear Nrf2 (**C**). Values are mean ± SD (*n* = 5). Different alphabetical letters on the bars (a–c) indicate statistically significant difference from each other (*p* < 0.05).

A key transcription factor of antioxidant and defense response, Nrf2, and one of its downstream proteins, HO-1, were highly expressed in B(a)P-treated lung tissues (Figure 6). Their expressions were increased by HJE more effectively than by NHJE. In addition, oral administration of HJE but not NHJE in mice increased the ratio of reduced to oxidized glutathione (GSH/GSSG) in lung homogenates (Supplementary Figure S3).

### 3.7. Oral Administration of HJE to Mice Increased Nrf2 and HO-1 Expression and Reduced Oxidative Stress in Liver Tissue

**Figure 7:**
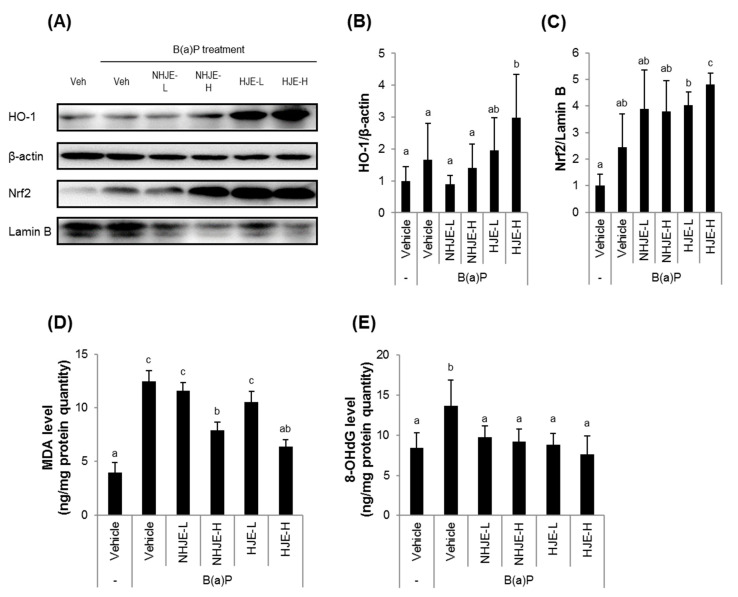
Oral administration of HJE increased Nrf2 and HO-1 expression and reduced oxidative stress in hepatic tissue. Liver tissue homogenates were used for analyses of expression levels of antioxidant proteins and oxidative stress markers. (**A**) Representative western blot images. (**B,C**) Quantitative data for cytosolic HO-1 (**B**) and nuclear Nrf2 (**C**). (**D**,**E**) Oxidative stress markers including MDA (**D**) and 8-OHdG (**E**) were quantified by ELISA. MDA, malondialdehyde. 8-OHdG, 8-hydoxydeoxyguanosine. Values are mean ± SD (*n* = 8). Different alphabetical letters presented on the bars (a–c) indicate statistically significant difference from each other (*p* < 0.05).

The relative quantities of nuclear Nrf2 and cytoplasmic HO-1 proteins were significantly higher in liver tissue from HJE-H-fed mice than from NHJE-fed or control mice (Figure 7A−C). In addition, oral administration of HJE reduced levels of oxidative stress markers, such as malondialdehyde (MDA) and 8-OHdG, in liver homogenates (Figure 7D−E).

## 4. Discussion

**Figure 8:**
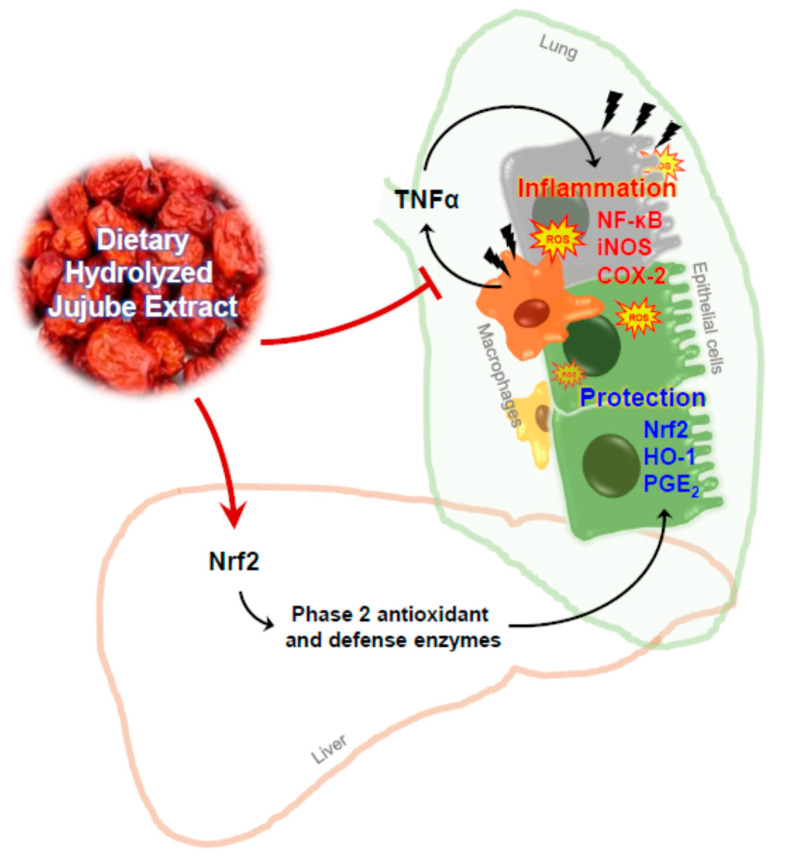
Schematic illustration of a potential mechanism for the anti-inflammatory activity of HJE.

Enzymatically hydrolyzed jujube extract was evaluated for its anti-inflammatory effect in a B(a)P-induced lung inflammation mouse model. Our findings demonstrated that (1) Viscozyme-mediated hydrolysis increased total phenolic content in an ethanolic extract of jujube fruit, (2) HJE inhibited TNFα production in TPA-differentiated THP-1 cells and inflammatory protein expression in A549 cells exposed to THP-1-conditioned media more effectively than did NHJE, and (3) oral supplementation with HJE for two weeks protected the lungs from B(a)P-induced morphological and histological damage, with decreased levels of inflammation-associated proteins in the lungs and increased levels of antioxidant/defense proteins in the lungs and liver. A graphical summary of the findings is presented in Figure 8.

Regardless of hydrolysis, jujube extracts used in this study were found to contain considerable amounts of total phenolics and flavonoids and demonstrate radical-scavenging abilities, which is consistent with previous reports [17,19,21]. HJE significantly promoted the transcription activity of the ARE-luciferase reporter gene and decreased production of NO, a signaling molecule regulating inflammatory processes, as shown by cell-based assays. In addition, HJE, but not NHJE, effectively inhibited TNFα secretion from TPA-differentiated THP-1 cells and suppressed inflammatory protein expression in A549 cells kept in THP-1-conditioned media. This suggests that the pre-treatment of jujube fruit with Viscozyme improved biological activity by increasing the bioavailability of bioactive compounds in jujube, an effect that may also be applicable to in vivo conditions.

To examine the in vivo anti-inflammatory effect of HJE compared with NHJE, a B(a)P-induced acute lung inflammation mouse model was employed [2,36,40]. B(a)P is the most common and toxic polycyclic aromatic hydrocarbon, an environmental pollutant, and reportedly the cause of health problems, such as redox imbalance, respiratory diseases, and cancer [41]. Benzo[a]pyrenediol-epoxide, which is metabolized from B(a)P by cytochrome P450 enzymes, provokes pulmonary inflammation by stimulating the NF-κB-mediated pathway in human lung fibroblasts [42]. Thus, B(a)P is widely used to generate a lung inflammation in mice [43,44,45]. Consistently, observations from this study demonstrated that a single administration of B(a)P to mice resulted in lung injury at morphological and histological levels. The elevation of pro-inflammatory cytokine levels in the blood samples was consistent with previous reports that showed B(a)P-induced deleterious consequences in multiple organs [41,45]. Moreover, oral supplementation of jujube extracts, both HJE and NHJE, reduced levels of NF-κB and its downstream cytoplasmic proteins, iNOS and COX-2, in the lungs while increasing expressions of Nrf2 and cytoplasmic HO-1 in lung and liver tissues. This suggests that dietary jujube extract exerts dual protective effects on both hepatic and pulmonary tissues from B(a)P-induced damage.

Intriguingly, our unpublished data demonstrated that Nrf2 and HO-1 protein expressions in A549 cells kept in THP-1-conditioned media were marginally altered by treatment with either HJE or NHJE. It implies that the stimulation of Nrf2-mediated antioxidant response in the lungs of the mice supplemented with jujube extract may be indirectly induced possibly through intestinal or hepatic metabolites. Specification and identification of biologically active metabolites produced from jujube extract in vivo will require additional study.

One of the critical findings of this study was that the biological effect of jujube extract was further enhanced when HJE was given to the mice compared with NHJE. It indicates that Viscozyme-mediated hydrolysis improved the beneficial potential of jujube fruits, likely by converting bioactive compounds in glycoside forms to aglycones and thereby increasing their bioavailability. A line of evidence has demonstrated that jujube contains a variety of bioactive substances [19,20]. Some sugars, terpenes, and phenolic acids have been reported as biologically functional components [21,24,26]. Rutin (quercetin-3-*O*-rutinoside), one of the major phenolics in jujube, has been demonstrated to possess antioxidative and anti-inflammatory activities [46,47,48].

Viscozyme includes a broad range of carbohydrase activities including β-glucanase, arabanase, and polygalacturonase, as described in previous reports [49,50]. Its cellulase and hemicellulase activities can degrade the polysaccharide components of plant cell walls [51,52]. HJE undergone Viscozyme-mediated hydrolysis was found to contain quercetin at levels 10.9-fold higher and total phenolic content at levels 1.9-fold higher than those of NHJE. These results are not surprising because rutin with β (1→6) glycosidic linkage can be broken by the enzyme and converted to quercetin. Furthermore, it is presumed that the β-glucanase and polygalaturonase activities have degraded cell walls and facilitated the extraction of phenolic compounds bound to cell-wall components. Considering the activity of gut microbiota and the protective effects of quercetin and rutin in the liver [53,54] and lungs against exogenous inflammatory agents [55,56,57,58,59,60], it is reasonable to assume that the hepatic and pulmonary protective effects of HJE from B(a)P insult are at least partially attributable to aglycones, including quercetin, released from glycosides during enzymatic treatment. However, further study is needed to identify the component(s) responsible for the anti-inflammatory activity of HJE. Whether the anti-inflammatory effect of HJE or its active components is mediated by the Nrf2/ARE signaling pathway or the NF-kB signaling pathway, or through crosstalk between both signaling pathways, is a fascinating question to address in the future.

## 5. Conclusions

This study demonstrated that dietary HJE can significantly ameliorate B(a)P-mediated lung inflammation and injury, possibly by inhibiting macrophage activity, suppressing NF-κB-mediated inflammatory protein expression in lung tissue, and stimulating Nrf2-mediated antioxidant/defense responses in the lung and liver. The findings would have implications for development of jujube-based functional foods beneficial to lung health, especially in situations where the respiratory system can be affected.

## Figures and Tables

**Table 1:** Total phenolics, rutin, and quercetin contents in jujube extracts ^1^.

	NHJE	HJE
Total phenolics (mg GAE ^2^/g dry weight)	6.73 ± 0.83 ^a^	12.50 ± 1.69 ^b^
Rutin (mg/g dry weight)	12.68 ± 6.06 ^a^	11.94 ± 4.99 ^a^
Quercetin (mg/g dry weight)	0.34 ± 0.59 ^a^	3.72 ± 2.54 ^b^

^1^ Values are expressed as means ± standard error of the mean (SEM) from three independent experimental sessions (*N* = 3). Different alphabetical letters by the values (a–b) indicate statistically significant difference from each other (*p* < 0.05). ^2^ GAE, gallic acid equivalent.

## Supplementary Materials

The following are available online at https://www.mdpi.com/2304-8158/9/8/1033/s1. Table S1: Total phenolic and flavonoid contents in jujube ethanolic extracts using various concentrations of ethanol in water. Table S2: Total phenolic content in 50% ethanol extract of jujube hydrolyzed with various enzymes. Figure S1: DPPH radical scavenging activity (A) and FRAP (B) of jujube extracts. Figure S2: Cytotoxicity of jujube extracts in THP-1 human monocytes (A) and A549 human lung epithelial cells (B). Figure S3. Dietary HJE increased the ratio of reduced to oxidized glutathione (GSH/GSSG) in lung homogenates.
