# Supplementary material for: In Vivo Anti-Inflammatory Potential of Viscozyme®-Treated Jujube Fruit
Source: Foods. 2020 Aug 1;9(8):1033. doi: 10.3390/foods9081033 (PMC7466189; doi:10.3390/foods9081033)
Supplement: Supplementary file 1 [file foods-09-01033-s001.pdf]

Supplementary information

For the manuscript entitled:
In Vivo Anti-inflammatory Potential of Viscozyme®-Treated Jujube Fruit

Includes

1) Supplementary Materials and Methods
2) Supplementary Figures: Fig. S1−3
3) Supplementary Tables: Table S1−2

1) Supplementary Materials and Methods

Determination of free radical scavenging capability and ferric-reducing antioxidant power

The antioxidant activities of NHJE and HJE were determined by the assays of 2,2-diphenyl-1-picrylhydrazyl (DPPH) radical scavenging activity and ferric-reducing antioxidant power (FRAP) as previously described [1-3]. α-tocopherol was used as a positive control.

Cell viability assay

cells were dispensed into a 96-well plate at a density of 5×10^3 cells/well, treated with jujube extract at the designated concentrations for 24 h, and assayed using the Cell Counting Kit (CCK-8; Dojindo Laboratories, Kumamoto, Japan) as previously described [2].

Measurement of reduced glutathione level

The lung tissue homogenates were used for analysis of the ratio of reduced glutathione (GSH) over oxidized glutathione (GSSG) level using glutathione detection kits (Cat # ADI-900-160; Enzo Life Sciences, Farmingdale, NY, USA). The analysis was performed according to the manufacture's instructions. Values were normalized to the quantity of total proteins.

References

1. Ozgen, M.; Reese, R.N.; Tulio, A.Z.; Scheerens, J.C.; Miller, A.R. Modified 2, 2-azino-bis-3-ethylbenzothiazoline-6-sulfonic acid (ABTS) method to measure antioxidant capacity of selected small fruits and comparison to ferric reducing antioxidant power (FRAP) and 2, 2 '-diphenyl-1-picrylhydrazyl (DPPH) methods. *J. Agric. Food Chem.* **2006**, *54*, 1151-1157.
2. Woo, Y.; Lee, H.; Jeong, Y.S.; Shin, G.Y.; Oh, J.G.; Kim, J.S.; Oh, J. Antioxidant Potential of Selected Korean Edible Plant Extracts. *BioMed Res. Int.* **2017**.
3. Benzie, I.F.; Strain, J.J. The ferric reducing ability of plasma (FRAP) as a measure of "antioxidant power": the FRAP assay. *Anal. Biochem.* **1996**, *239*, 70-76.

2) Supplementary Figures

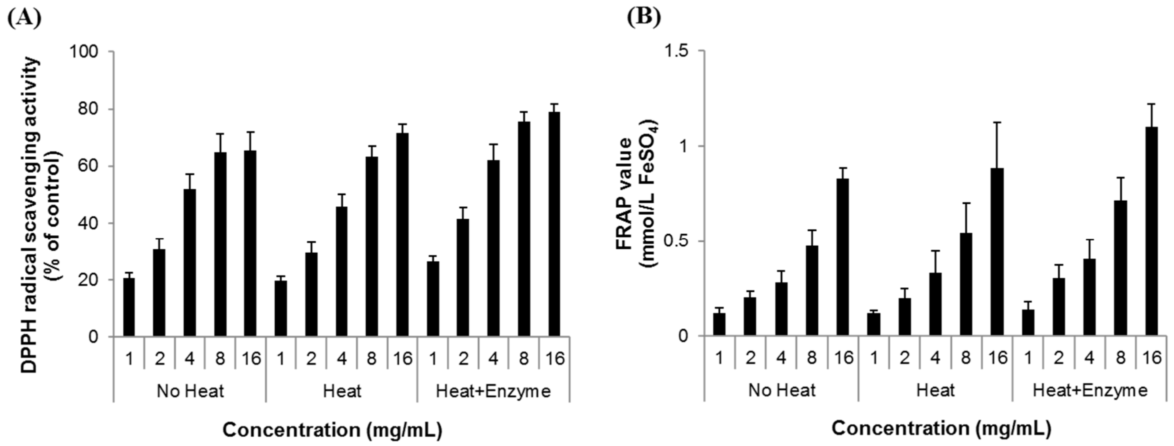

Supplementary Figure S1. DPPH radical scavenging activity (A) and FRAP (B) of jujube extracts. Both extract samples (HJE and NHJE) showed a concentration-dependent antioxidant capability; in particular, HJE was more effective than NHJE. Values are presented as mean ± SD (N = 3). NHJE, non-hydrolyzed jujube extract. HJE, hydrolyzed jujube extract.

(A) (B)

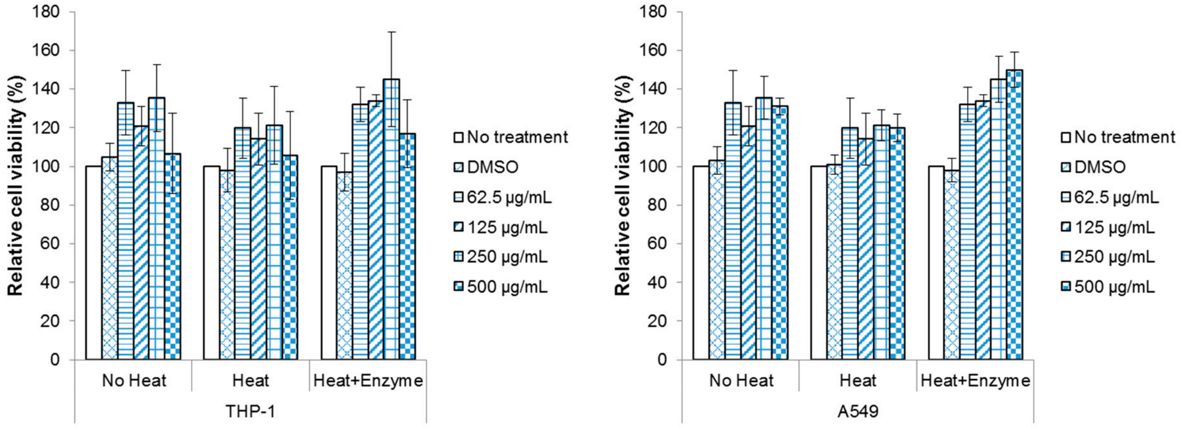

Supplementary Figure S2. Cytotoxicity of jujube extracts in THP-1 human monocytes (A) and A549 human lung epithelial cells (B). Both types of cells were treated with jujube extracts at the designated concentrations. The both extracts were non-toxic at ≤ 500 μg/mL. Values are presented as mean ± SEM (n = 3).

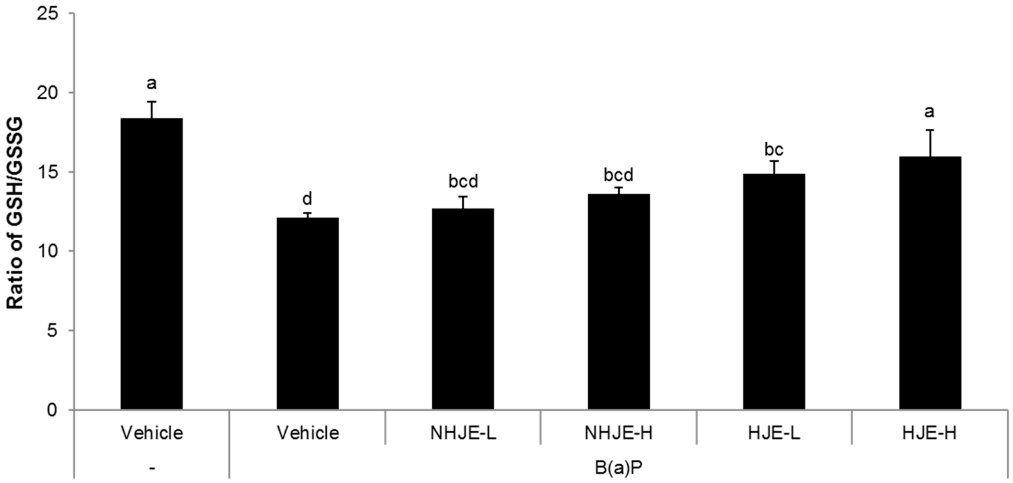

Supplementary Figure S3. Dietary HJE increased the ratio of reduced to oxidized glutathione (GSH/GSSG) in lung homogenates. Values are presented as mean ± SD (n = 5). Different alphabetical letters presented on the bars indicate statistically significant difference from each other ($p < 0.05$).

3) Supplementary Tables

Supplementary Table S1. Total phenolic and flavonoid contents in jujube ethanolic extracts using various concentrations of ethanol in water[1)]

	Ethanol concentration of extraction solvent (v/v) in water				
	0%	20%	50%	80%	100%
Total phenolic content (mg GAE[2)]/g DW[3)])	2.01 ± 0.88[a]	4.02 ± 0.19[a]	6.73 ± 0.83[b]	5.33 ± 1.20[b]	5.88 ± 1.59[b]
Total flavonoid content (mg QE[4)]/g DW)	2.35 ± 0.09[a]	2.48 ± 0.09[a]	3.61 ± 0.12[b]	2.64 ± 0.12[a]	2.31 ± 0.03[a]

[1)]Values are expressed as means ± SD ($n = 3$). Different alphabetical letters presented on the bars indicate statistically significant difference from each other ($p < 0.1$).
[2)]GAE, gallic acid equivalent
[3)]DW, dry weight of ethanol extract
[4)]QE, quercetin equivalent

Supplementary Table S2. Total phenolic content in 50% ethanol extract of jujube hydrolyzed with various enzymes[1)]

Enzyme used	Total phenolic content (mg GAE[2)]/g DW[3)])
No Enzyme	18.59 ± 10.26
Viscozyme (β-glucanase, cellulase, hemicellulase)	27.09 ± 11.64
Fungamyl (α-amylase)	14.20 ± 2.95
AMG (α-glucosidase)	15.38 ± 4.67
Viscozyme + Fungamyl	17.46 ± 3.01
Viscozyme + AMG	18.89 ± 4.86

[1)]Values are expressed as means ± SD (*n* = 3).
[2)]GAE, gallic acid equivalent
[3)]DW, dry weight of ethanol extract
